# Different responses to glucose overload between two strains of largemouth bass (*Micropterus salmoides*)

**DOI:** 10.3389/fphys.2022.1010633

**Published:** 2022-09-28

**Authors:** Caixia Lei, Yujing Xie, Hongmei Song, Peng Jiang, Jinxing Du, Shengjie Li

**Affiliations:** ^1^ Key Laboratory of Tropical and Subtropical Fishery Resources Application and Cultivation, Ministry of Agriculture and Rural Affairs, Pearl River Fisheries Research Institute, Chinese Academy of Fishery Sciences, Guangzhou, Guangdong, China; ^2^ Key Laboratory of Fishery Drug Development, Ministry of Agriculture and Rural Affairs, Pearl River Fisheries Research Institute, Chinese Academy of Fishery Sciences, Guangzhou, Guangdong, China; ^3^ College of Fisheries and Life Science, Shanghai Ocean University, Shanghai, China

**Keywords:** glucose overload, gene expression, transcriptional analysis, genotype, *Micropterus salmoides*

## Abstract

In order to improve the glucose utilization capacity of largemouth bass (*Micropterus salmoides*), responses to glucose overload between two strains (Y: breeding strain; W: wild strain) were compared at 0, 6, 12, and 24 h after glucose injection (1.67 g/kg). The data revealed that plasma glucose in the Y strain (<12 h) recovered faster than in the W strain (12 h), with the Y strain secreted more insulin within 6 h post-injection. Triglyceride (TG) and low-density lipoprotein-cholesterol (VLDL-CH) content in the Y strain increased, peaking at 12 h, then decreased, whereas the W strain’s TG content was not affected and VLDL-CH content decreased. The hepatic and muscular fatty acid synthetase, liver x receptor-1, and sterol regulatory element-binding protein expressions were consistent with the TG content change. Both strains’ liver and muscle glycogen contents exhibited similar trends to that of the glycogen synthase gene—increasing, then declining, and peaking at 6 and 12 h. The expression levels of hepatic and muscular phosphofructokinase and pyruvate kinase in the Y strain increased, peaking at 12 h. In the W strain, they were suppressed and reached the minimum at 24 h. The mRNA levels of hepatic and muscular phosphoenolpyruvate carboxykinase and glucose-6-phosphatase were enhanced and peaked at 24 h in both strains, hepatic isocitrate dehydrogenase-1, and α-ketoglutarate dehydrogenase complex expression increased after declining, peaking at 12 and 24 h. Two genes in the W strain’s muscles showed a similar trend. Both strains’ transcriptome results identified seven common functional genes for resistance to hyperglycemia that were involved in the circadian rhythm pathway, which is a suggested key pathway for coping with hyperglycemia. Furthermore, 48 differential genes were identified between the two strains, and these genes were enriched in the TGF-beta and cell cycle signaling pathways, indicating that these pathways may be key factors affecting the differential responses to glucose overload. We conducted a comprehensive comparison of glucose overload molecular responses between two strains of *M. salmoides*, and the results can provide a promising strategy to improve the glucose utilization capacity of *M. salmoides* based on advantageous pre-existing traits.

## Introduction

Protein is one of the major energy sources for fish, and constitutes high proportions of fish feed composition. Contemporarily, fish meal is recognized as a top-quality protein source in aquafeeds, although statistics show that the total marine catches have been relatively stable since the mid-2000s (FAO, 2020), indicating that limited fish meal is expected to be incapable to meet the needs of rapid developing aquaculture, especially those of carnivorous fish farming. Plant proteins (e.g., soybean meal and cottonseed meal) that contain relatively high levels of carbohydrates, have already been widely used as the fish meal substitutes. However, teleost fish, particularly carnivorous fish, are commonly regarded as “born diabetic” (Moon, 2001). High glucose levels in these fish have been demonstrated to cause mitochondrial dysfunction, decreased specific growth rate, and suppressed immune functions (Liu et al., 2012; Li et al., 2019; Liu et al., 2020), thereby requiring the restriction of dietary plant proteins. Nevertheless, carbohydrates are the most economical energy source in aquafeeds and are regarded as a non-nitrogenous energy source. Its addition is cost-saving, resource-saving, and environmentally friendly. Currently, there are numerous studies focused on maximizing the added level of carbohydrates in the feed through nutrition, such as adjusting the dietary carbohydrate to lipid ratio (Taj et al., 2020), dietary carbohydrate to protein ratio (Betancor et al., 2018), and carbohydrate source (Tian et al., 2010). However, it is fundamentally and applied to improve the carbohydrate utilization capacity of farmed fish *via* genetic breeding.

The glucose turnover rate in fish is inefficient compared with that in mammals (Weber and Zwingelstein, 1995), although all carbohydrate metabolic enzymes identified in fish (Polakof et al., 2012). Initially, the theory of insufficient secretion of a glucose scavenger (insulin), which leads the fish to have “diabetic constitution” was proposed; Later, hypotheses suggested a lack of insulin receptors and glucose transporters that resulted in inefficient responses to insulin and inefficient absorption of glucose, thereby causing fish to have poor dietary glucose utilization capacities (Kamalam et al., 2017). Nevertheless, these assumptions were rejected (Mommsen and Plisetskaya, 1991; Navarro et al., 2002; Díaz et al., 2007; Díaz et al., 2009). Until now, the mechanism underlying glucose intolerance in fish has not been thoroughly explained. Furthermore, the response of fish to a glucose load is species-specific, and it has been reported that rate-limiting enzymes involved in glycolysis and gluconeogenesis are not affected by dietary carbohydrate levels. Alternatively, the European sea bass (*Dicentrachus labrax*) reveal a metabolic adaptation of lipogenesis to dietary carbohydrate levels (Moreira et al., 2008), while the gilthead sea bass’s (*Sparus aurata*) glycolytic enzymes were enhanced by a high-carbohydrate diets, which also depressed key gluconeogenic enzymes (Panserat et al., 2000; Panserat et al., 2002; Fernandez et al., 2007). In rainbow trout (*Oncorhynchus mykiss*), accelerated glycolysis and glycogen synthesis after high glucose fluxes were observed, whereas the lipogenesis and gluconeogenesis pathways were not affected (Enes et al., 2009).

The responses to glucose overload are various in different strains of fish. In the two strains of *O. mykiss*, the muscular glycogen content was higher in the strain prone to more body fat content than in the leaner strain after glucose overload. Moreover, higher expressions of the liver glycolytic enzyme gene and muscle fatty acid oxidation enzymes were observed in the fattier strain, indicating that the fattier strain of *O. mykiss* may have better glucose tolerance than the leaner strain (Jin et al., 2014). Similarly, differential glucose metabolism has been reported between the natural wild strain (DT strain) and selected strain with faster growth (A strain) of Gibel carp (*Carassius gibelio*), where enhanced mRNA levels of hepatic glucokinase (*gk*), acyl-CoA oxidase 3, and muscular carnitine palmitoyl transferase 1 isoform-a were observed in the DT strain after insulin injection. The opposite results were found for strain A (Jin et al., 2017).

Largemouth bass (*Micropterus salmoides*) is now a very popular food-fish in China. As a carnivorous fish, the *M. salmoides* has a limited glucose utilization capacity, which poses a difficulty in farms as fish meal is an essential component of its diet (Amoah et al., 2008). Therefore, improving its glucose utilization capacity is important for the sustainable development of the aquaculture industry. Herein, we selected a new *M. salmoides* strain (Y strain) with faster growth and better receptiveness to pelleted feeds that have been widely accepted by Chinese fishermen (Li et al., 2018). In comparison, a wild strain (W strain), which grows slowly and prefers frozen meat bait, is relatively unpopular. Considering that the Y strain’s excellent economical traits may be related to carbohydrate utilization capacity, this study was performed to compare the difference in carbohydrate utilization between the two strains and investigate the mechanism behind this difference. The data presented herein provide new opportunities for enhancing *M. salmoides* carbohydrate utilization capacity based on advantageous pre-existing traits.

## Materials and methods

### Fish preparation and sampling

The experimental *M. salmoides* (initial weight of Y strain: 49.38 ± 4.45 g; initial weight of W strain: 49.08 ± 4.39 g) were provided by Liang’s aquatic seed Industry Co., Ltd., Foshan, Guangdong, China. Then, 210 fish were evenly and randomly assigned into 30 plastic buckets (0.18 m^3^/bucket, 15 buckets per strain, 7 fish/bucket). After being fasted for 24 h and anesthetized with 2-phenoxyethanol (0.01%, w/v; Sigma-Aldrich LLC., Shanghai, China), the fish were intraperitoneally injected with glucose (1.67 g/kg; Sigma-Aldrich LLC., Shanghai, China). The fish were sampled at basic-0 (injected with isometric normal saline), 0, 6, 12, and 24 h after injection, and 4 fish were randomly sampled from each bucket. Considering that there are 3 buckets at each time point per strain, a total of 12 fish were sampled at each time point per strain. Blood was quickly collected in anticoagulant tubes (Solarbio Science and Technology Co., Ltd., Beijing, China), and plasma was prepared by centrifugation at 2,500 rpm for 10 min. The liver and muscle tissues were also sampled and stored along with the plasma at −80°C.

### Plasma parameters detection

Plasma glucose (Solarbio Science and Technology Co., Ltd., Beijing, China) and triglyceride (TG) contents (Applygen Technologies Inc., Beijing, China) were detected using commercial kits according to the principle that colored compounds are finally produced through a series of enzymatic reactions, and the optical density values of colored compounds could be used as the measuring index. The absorbance of the reaction mixture was measured at 505 nm (glucose) and 550 nm (TG), respectively. The plasma very low-density lipoprotein-cholesterol (VLDL-CH) and insulin levels were determined by ELISA, and the kits were obtained from Shanghai Enzyme-linked Biotechnology Co., Ltd., Shanghai, China. All procedures were performed according to the manufacturers’ instructions, and a microplate reader (Thermo Fisher Scientific Co., Ltd., Shanghai, China) was used to measure the absorbance.

### Liver triglyceride content analysis

Liver TG content was measured using a commercial kit (Applygen Technologies Inc., Beijing, China) following the manufacturer’s recommendation. The liver samples were first homogenized in the lysate (1:20, m/v), followed by incubated at 70°C for 10 min. Afterwards, the mixture was centrifuged at 2,000 rpm for 5 min and the upper liquid was obtained for TG content analysis. The absorbance was measured at 550 nm using a microplate reader (Thermo Fisher Scientific Co., Ltd., Shanghai, China).

### Glycogen assay

Liver and muscle glycogen contents were determined using a commercial kit purchased from Solarbio Science and Technology Co., Ltd., (Beijing, China). According to the instructions provided by the manufacturer, the samples were homogenized in precooled distilled water at a ratio of 1:8 (w/v) and boiled for 10 min before the test. The supernatants were obtained for glycogen assays after cooling and centrifugation (8,000 × g, 25°C, 10 min). Glycogen content was determined by measuring the absorbance at 505 nm using a microplate reader (Thermo Fisher Scientific Co., Ltd., Shanghai, China).

### RNA extraction and quantitative real-time polymerase chain reaction

Total RNA samples from the liver and muscle were isolated using the TRIzol reagent (Takara Biomedical Technology Co., Ltd., Dalian, China). cDNA was synthesized using a reverse-transcription kit (Takara Biomedical Technology Co., Ltd., Dalian, China) after the RNA samples were quantified using a Thermo NanoDrop Spectrophotometer (Thermo Fisher Scientific Co., Ltd., Shanghai, China), and quality was tested using a 1% agarose gel electrophoresis. The specific operation steps were recommended by the reagent manufacturer. The gene expression was amplified in triplicate using the Step-One-plus real-time PCR system (Applied Biosystems, Foster City, CA, United States) according to our previous study (Lei et al., 2022). The amplification mixture was comprised of 1 μl (25 ng) cDNA template, 0.8 μl each of forward and upstream primers, 10 μl 2 × Power SYBR™ Green PCR Master Mix (Thermo Fisher Scientific Co., Ltd., Shanghai, China), and 7.4 μl sterilized ultrapure water. The PCR conditions were initialed at 95°C for 5 min, followed by 40 cycles of denaturation at 95°C for 15 s, annealing at a specific temperature (Table 1) for 1 min, and extension at 72°C for 1 min. Ultimately, a melting curve was obtained to confirm that only the signal product was amplified. Two negative controls (no cDNA and non-reverse transcribed RNA) were used to confirm that only cDNA was quantified. The geometric mean of house-keeping genes (beta-actin and glyceraldehyde-3-phosphate dehydrogenase) was used to normalized gene expression. The results were calculated using the comparative CT method (2^−ΔΔCt^) (Livak and Schmittgen, 2001; Pfaffl, 2001) and expressed as the fold change of the of control group.

**TABLE 1:** Primers used for qRT-PCR.

Functional classification	Gene	Forward and reverse primer (5′-3′)	Annealing temperature (°C)	Product size
Glycolysis	*gk*	GGG​GAT​GGA​AAG​CAA​ATC​TAC​AAT; CAC​ACA​TAC​GAG​CAG​AGC​GAG​T	61	121 bp
	*pfk*	GGC​TGG​GCT​ATG​ATA​CAA​GAG​TGA; CTC​CAT​TAG​AGG​CAG​ACG​AAC​A	61	194 bp
	*pk*	ATC​GCC​ATG​CCT​CAT​CCA​AA; CAG​ATG​ATG​CCA​GTG​TTG​CG	58	155 bp
Gluconeogenesis	*pepck*	CAT​CAA​CCC​GGA​GAA​TGG​CT; CAC​AGG​GTT​CGC​CAT​CTT​CT	61	224 bp
	*g6p*	CTG​GGT​GCA​TCA​TCA​GCT​CT; TCT​TGC​AGA​AGG​ACA​GCA​GG	61	102 bp
	*fbp*	GAC​AAC​CCT​GCT​CAA​CTC​CA; AAG​CAC​ACA​GGA​GGT​GAA​GG	61	193 bp
TG synthesis	*fas*	ACC​ACA​CCC​TCA​TAC​CGA​CT; GGG​AGA​GTG​TCC​ACA​ACA​CA	58	220 bp
	*lxr-1*	CTC​CCA​CCC​CAA​TGA​CTT​CC; GCA​GGC​CCC​TTC​TTT​CTC​TT	61	165 bp
	*srebp*	TTC​CTC​TCC​CTC​AAC​CCT​C; AAC​CCC​AGA​AAC​CAG​AAT​ACC	56	195 bp
Glycogen synthesis	*gs*	TCA​CAG​CCA​TTG​AGG​CAG​AG; GTA​AAA​GTG​CCC​CCT​GAC​GA	61	158 bp
Krebs cycle	*icdh-1*	CAG​ACC​GCA​ACA​GAG​GAA​CT; GCC​AGA​GCA​GAG​TCA​ATG​GT	61	213 bp
	*kgdc*	TCT​CAC​CGT​TCC​CGT​TTG​A; CCT​GGT​TCT​TGT​GCT​CCT​CCT	61	93 bp
House-keeping gene	*β-actin*	CTT​TCC​TCG​GTA​TGG​AGT​CTT​G; GGT​CAG​CGA​TTC​CAG​GGT​A	58	141 bp
	*gapdh*	CTT​CGT​CAT​TCC​CCG​CTA​CT; CGG​GGA​ATC​GAG​GGT​TGT​AT	61	109 bp

Accession number: MT431528.1 (glucokinase, *gk*); MT431526.1 (pyruvate kinase, *pk*); XM_038733827.1 (fructose bisphosphatase, *fbp*); XM_038697432.1 (glycogen synthase, *gs*); XM_038701842.1 (isocitrate dehydrogenase-1, *icdh-1*); XM_038735140.1 (fatty acid synthetase, *fas*); XM_038700506.1 (liver X receptor-1, *lxr-1*); XM_038699585.1 (sterol regulatory element-binding protein, *srebp*); XM_038712008.1 (glyceraldehyde-3-phosphate dehydrogenase, *gapdh*); MH018565.1 (beta-actin, *β-actin*). The sequences of phosphofructokinase (*pfk*), phosphoenolpyruvate carboxykinase (*pepck*), glucose-6-phosphatase (*g6p*), and alpha-ketoglutarate dehydrogenase complex (*kgdc*) have been cloned and will be published in our another article.

### Transcriptome sequencing

Liver RNA samples from the 6 and 12 h time groups were selected from the extracted RNA samples and transcriptomes were sequenced by Majorbio Bio-pharm Biotechnology Co., Ltd., (Shanghai, China) on the Illumina sequence platform. At each time point, four samples from one bucket were mixed in equal volumes, and 12 samples (6 samples per strain) were obtained. After total RNA was separated and fragmented, cDNA was synthesized using a SuperScript double-stranded cDNA synthesis kit (Invitrogen Life Technology Co., Ltd., CA, United States). After being end-repaired, phosphorylated, and added with “A” base, 200–300 bp cDNA fragments were selected and for subsequent amplification with 15 cycles. Finally, 12 libraries were prepared and sequenced on an Illumina Novaseq 6000 (Illumina Inc., San Diego, CA, United States).

### RNA-seq analysis and validation

Raw RNA-seq data were filtered using the SeqPrep software (https://github.com/jstjohn/SeqPrep). The reads containing adapters, reads with over 10% unknown nucleotides, and reads with >50% low-quality bases with Q-value ≤ 20% were removed. The remaining reads were assembled using the *M. salmoides* genome (https://www.ncbi.nlm.nih.gov/genome/10791? genome_assembly_id = 1468587) and annotated with reference to the six databases. The web sites of these databases are shown in Supplementary Table S1 in the Supplementary Material. Genes with *p* < 0.05 and |log2FC| ≥ 1 (FC: fold change between groups) were selected and GO and KEGG analyses were then performed (Xie et al., 2011). Seven unigenes were also randomly selected and quantified using qRT-PCR to validate the results of the significant gene analysis, and the primer sequences are displayed in Supplementary Table S2 in the Supplementary Material. Transcripts amplification and analysis were performed as described above.

### Statistical analysis

All data are presented as mean ± standard deviation (SD). Plasma parameters were analyzed using two-way analysis of variance. The liver TG content, tissue glycogen content and gene expression were subjected to a one-way analysis of variance (ANOVA). The scheffe multiple comparison test was used to determine the statistical significance among the means. Tests for normality and homoscedasticity were performed prior (Kamalam et al., 2012) to formal statistical analysis (SPSS 18.0, Chicago, IL, United States).

## Results

### Plasma parameters after glucose tolerance test

The data presented in Table 2 depict the effect of glucose injection on plasma glucose, TG, VLDL-CH, and insulin levels. The data reveal that the glucose level decreased with increasing time and reached the lowest level at 12 h in the Y strain and 24 h in the W strain. The TG content increased after declining in the Y strain and peaked at 12 h, whereas no significant difference was found in the W strain’s TG content. The highest VLDL-CH content was observed at 6 and 12 h in the Y strain, it decreased and reached a minimum at 24 h in the W strain. The insulin level was increased after declining in both strains, and the highest level was observed at 6 h, followed by 0, 12, and 24 h. Notably, these plasma parameters varied between the two strains. The plasma glucose level in the Y strain was markedly lower than in the W strain from 6 h onwards, and the Y strain was found to have a higher TG content than the W strain at 12 h. Meanwhile, a higher VLDL-CH content was also observed at 6 and 12 h in the Y strain than in the W strain. Insulin levels in the Y strain were also higher than those in the W strain at 0 and 6 h.

**TABLE 2:** Plasma parameters. Data are expressed as mean ± SD (*n* = 12). Different letters indicate significant differences (*p* < 0.05). Lowercase letters mean the differences in the same strain at different time points; Capital letters mean the differences between the two strains at the same time point.

	Glucose (mM)			TG (mM)		
Time (h)	Y	W	*p* (W ✕ Y)	Y	W	*p* (W ✕ Y)
Basic-0	8.28 ± 0.15	8.42 ± 1.74	0.819	1.45 ± 0.22	1.31 ± 0.18	0.491
0	18.07 ± 0.15^a^	18.17 ± 0.45^a^	0.842	1.38 ± 0.44^b^	1.22 ± 0.18	0.372
6	10.67 ± 1.24^bB^	13.31 ± 0.97^bA^	0.000	1.36 ± 0.39^b^	1.58 ± 0.19	0.254
12	4.33 ± 1.06^cB^	7.87 ± 0.94^cA^	0.000	2.89 ± 0.93^aA^	1.50 ± 0.20^B^	0.000
24	4.19 ± 0.57^cB^	5.33 ± 0.9^dA^	0.035	1.14 ± 0.13^b^	1.18 ± 0.13	0.196
	VLDL-CH (ng/ml)			Insulin (μg/L)		
Time (h)	Y	W	*p* (W ✕ Y)	Y	W	*p* (W ✕ Y)
Basic-0	101.97 ± 1.83	103.58 ± 1.70	0.271	4.55 ± 0.53	3.89 ± 0.71	0.112
0	104.03 ± 1.70^b^	101.88 ± 1.59^a^	0.358	27.45 ± 2.32^bA^	14.57 ± 1.88^bB^	0.000
6	113.28 ± 1.83^aA^	102.97 ± 1.70^abB^	0.000	183.24 ± 8.79^aA^	154.29 ± 4.34^aB^	0.000
12	114.78 ± 1.83^aA^	106.39 ± 1.06^aB^	0.001	16.29 ± 1.04^c^	17.21 ± 1.35^c^	0.783
24	98.12 ± 1.59^b^	93.88 ± 1.59^b^	0.085	1.05 ± 0.08^d^	0.86 ± 0.12^d^	0.224

TG, triglyceride; VLDL-CH, low-density lipoprotein-cholesterol.

### Liver triglyceride content

**FIGURE 1:**
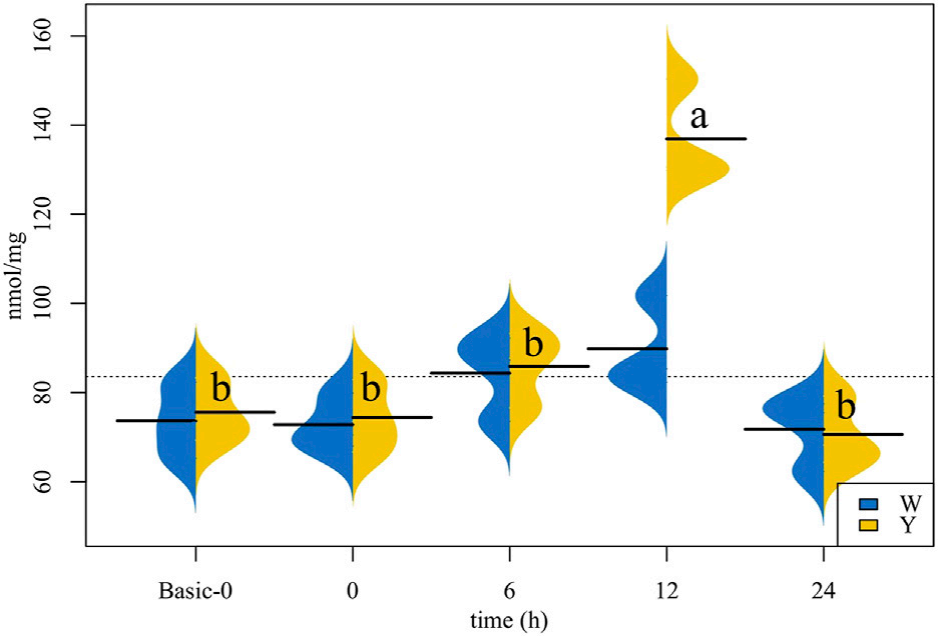
Triglyceride (TG) content in the liver (*n* = 12). The values corresponding to the horizontal lines are the mean of the data. Different letters indicate significant differences (*p* < 0.05).

According to the data depicted in Figure 1, the liver TG content in the Y strain increased first and then decreased, the TG content at 12 h time group was higher than the other time groups. Although the liver TG content in the W strain shared the same change trend as the Y strain, no statistical differences were found.

### Tissue glycogen content

**FIGURE 2:**
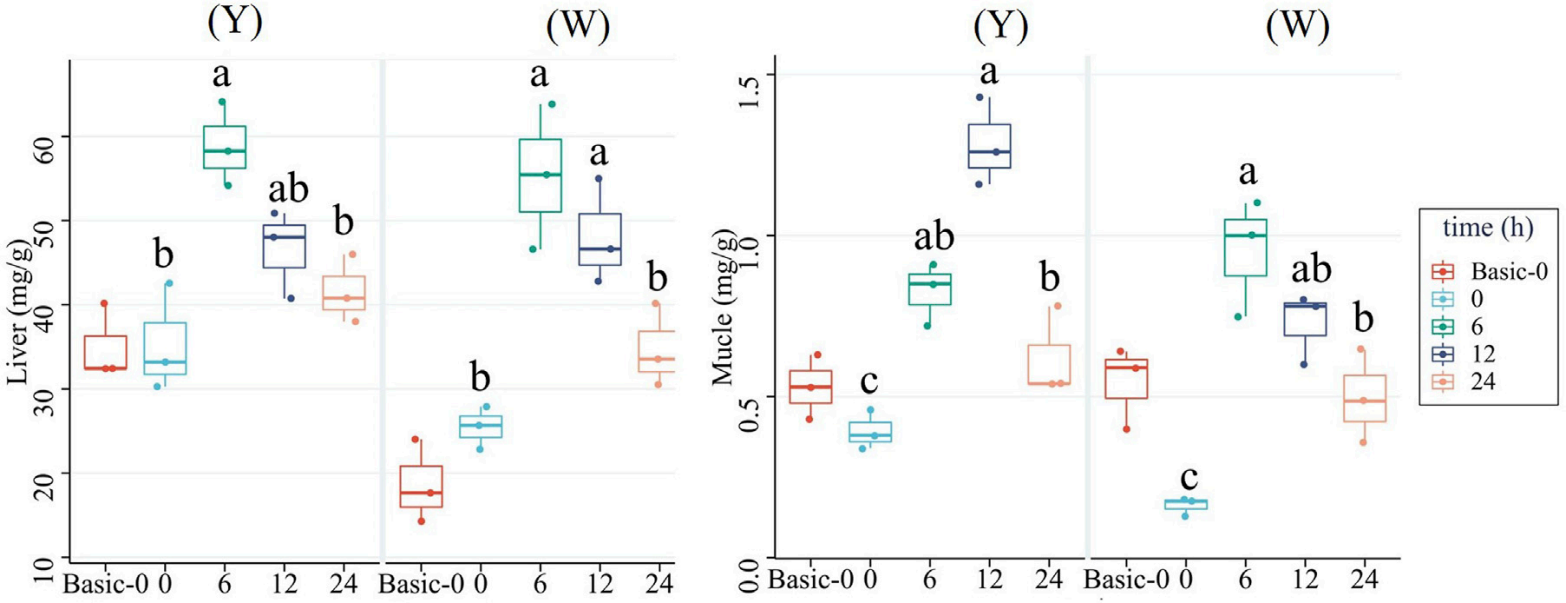
Glycogen contents in the liver and muscle. Data are expressed as mean + SD (*n* = 12). Different letters indicate significant differences (*p* < 0.05).

As shown in Figure 2, both strains’ liver and muscle glycogen contents shared the same trend of increasing first and then decreasing, with higher contents observed at 6 and 12 h. Besides, the muscle glycogen content at 24 h was higher than that at 0 h.

### Expression of hepatic genes involved in glucose overload response

**FIGURE 3:**
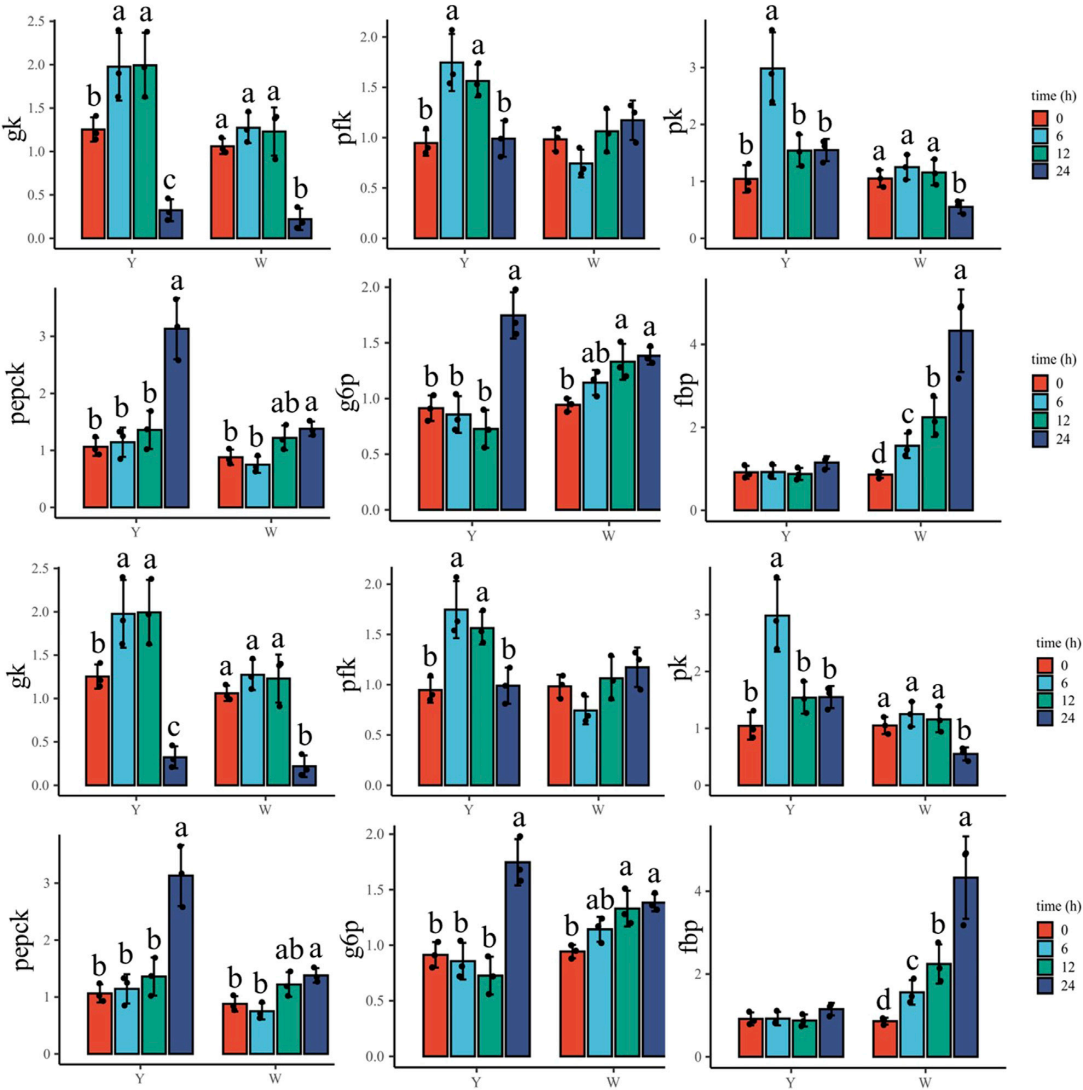
Expression of hepatic genes involved in glucose overload response. Data are expressed as mean + SD (*n* = 12). Different letters indicate significant differences (*p* < 0.05). *gk*, glucokinase; *pfk*: phosphofructokinase; *pk*, pyruvate kinase; *pepck*, phosphoenolpyruvate carboxykinase; *g6p*, glucose-6-phosphatase; *fbp*, fructose bisphosphatase; *fas*, fatty acid synthetase; *lxr-1*, liver X receptor-1; *srebp*, sterol regulatory element-binding protein; *icdh-1*, isocitrate dehydrogenase-1; *kgdc*, alpha-ketoglutarate dehydrogenase complex; *gs*, glycogen synthase.

To understand the response to glucose overload in the liver post high-glucose challenge, the expression of related genes was detected (Figure 3). For glycolysis, the two strains shared a similar *gk* expression tendency, which increased then decreased. The lowest mRNA levels were observed at 24 h. The phosphofructokinase (*pfk*) gene showed higher expressions at 6 and 12 h in the Y strain, whereas no significant differences were observed in the W strain. The transcription of pyruvate kinase (*pk*) in the Y strain reached a maximum at 6 h, but in the W strain gradually decreased and reached a minimum at 24 h. The genes involved in gluconeogenesis—phosphoenolpyruvate carboxykinase (*pepck*) and glucose-6-phosphatase (*g6p*)—gradually increased in expression in the Y strain, and were also upregulated in the W strain. Specifically, they exhibited higher gene expression after 12 h until peaking at 24 h. Although fructose bisphosphatase (*fbp*) expression remained stable in the Y strain, it was increased with time in the W strain. The mRNA levels of fatty acid synthetase (*fas*), liver x receptor (*lxr*)-1, and sterol regulatory element-binding protein (*srebp*) exhibited diverse regulation in the two strains. In the Y strain, both *fas* and *lxr-1* were upregulated and stabilized at 12 h, at which time, *srebp* expression was significantly higher than in the other time groups. There were no notable differences in *fas* and *lxr-1* expression observed among the time groups in the W strain, and lower *srebp* expression was found at 24 h. With the increasing glucose challenge time, the general trend of isocitrate dehydrogenase (*icdh*)-1, α-ketoglutarate dehydrogenase complex (*kgdc*), and glycogen synthase (*gs*) gene expression increased after declining in both strains. For *icdh-1*, the highest and lowest mRNA levels were observed at 12 and 0 h, respectively. *Kgdc* in the strain Y shared a similar expression pattern, although it was still highly expressed at 24 h in the W strain. For *gs* gene, the highest expression was appeared at 12 h in both strains.

### Expression of muscular genes involved in glucose overload response

**FIGURE 4:**
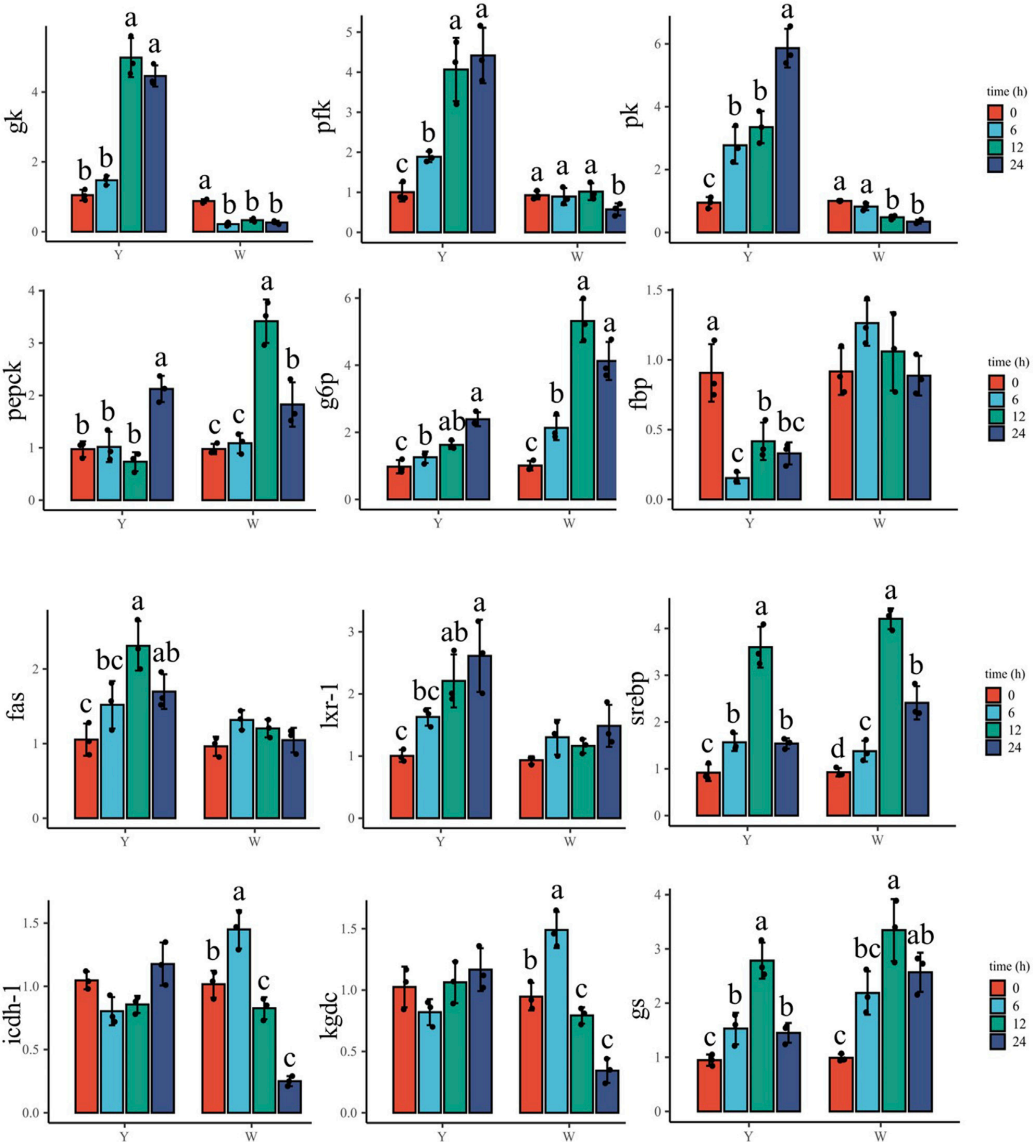
Expression of muscular genes involved in glucose overload response. Data are expressed as mean + SD (*n* = 12). Different letters indicate significant differences (*p* < 0.05). *gk*, glucokinase; *pfk*, phosphofructokinase; *pk*, pyruvate kinase; *pepck*, phosphoenolpyruvate carboxykinase; *g6p*, glucose-6-phosphatase; *fbp*, fructose bisphosphatase; *fas*, fatty acid synthetase; *lxr-1*, liver X receptor-1; *srebp*, sterol regulatory element-binding protein; *icdh-1*, isocitrate dehydrogenase-1; *kgdc*, alpha-ketoglutarate dehydrogenase complex; *gs*, glycogen synthase.

As shown in Figure 4, *gk* increased in response to the glucose challenge, and its highest mRNA level was observed at 12 and 24 h in the Y strain. However, it decreased in the W strain, with the highest expression at 0 h. *Pfk* and *pk* genes were enriched with time in the Y strain, and the highest and lowest expressions appeared at 24 and 0 h, respectively. In the W strain, the two genes decreased throughout the time samples and reached their lowest levels at 24 h. The gene expression of *pepck* increased and peaked at 24 h in the Y strain, while a higher *pepck* expression was also observed in the W strain, which was lower than that at 12 h. The *g6p* gene showed upregulation in both strains, and the highest mRNA levels were found at 12 and 24 h. *Fbp* gene transcription was suppressed in the Y strain and was the highest at 0 h, followed by 12 and 24 h. No significant difference was observed in the W strain *fbp* expression among the time groups. Among the genes involved in TG synthetase, the *fas* gene in the Y strain was elevated and peaked at 12 h, whereas no statistical differences in *fas* gene expression were observed in the W strain. *Lxr-1* showed a trend similar to that of *fas*. It increased until 12 h in the Y strain, while it showed no significant differences among the W strain’s time groups, although there was a slight upward trend. In both strains, *srebp* showed a similar trend of increasing after declining. The highest expression was found at 12 h and the lowest expression was observed at 0 h. Genes involved in tricarboxylic acid cycle (*icdh-1* and *kgdc*) and *gs* showed an alike upward trend and then downward trend in the W strain, in which the most abundant mRNA levels of *icdh-1* and *kgdc* were observed at 6 h followed by at 0 h. The highest and lowest of *gs* transcription were found at 12 and 0 h, respectively. In the Y strain, *icdh-1* and *kgdc* were not affected. Meanwhile, the *gs* gene first increased and then decreased, and peaked at 12 h, followed by 6 and 24 h.

### Transcriptome analysis

**FIGURE 5:**
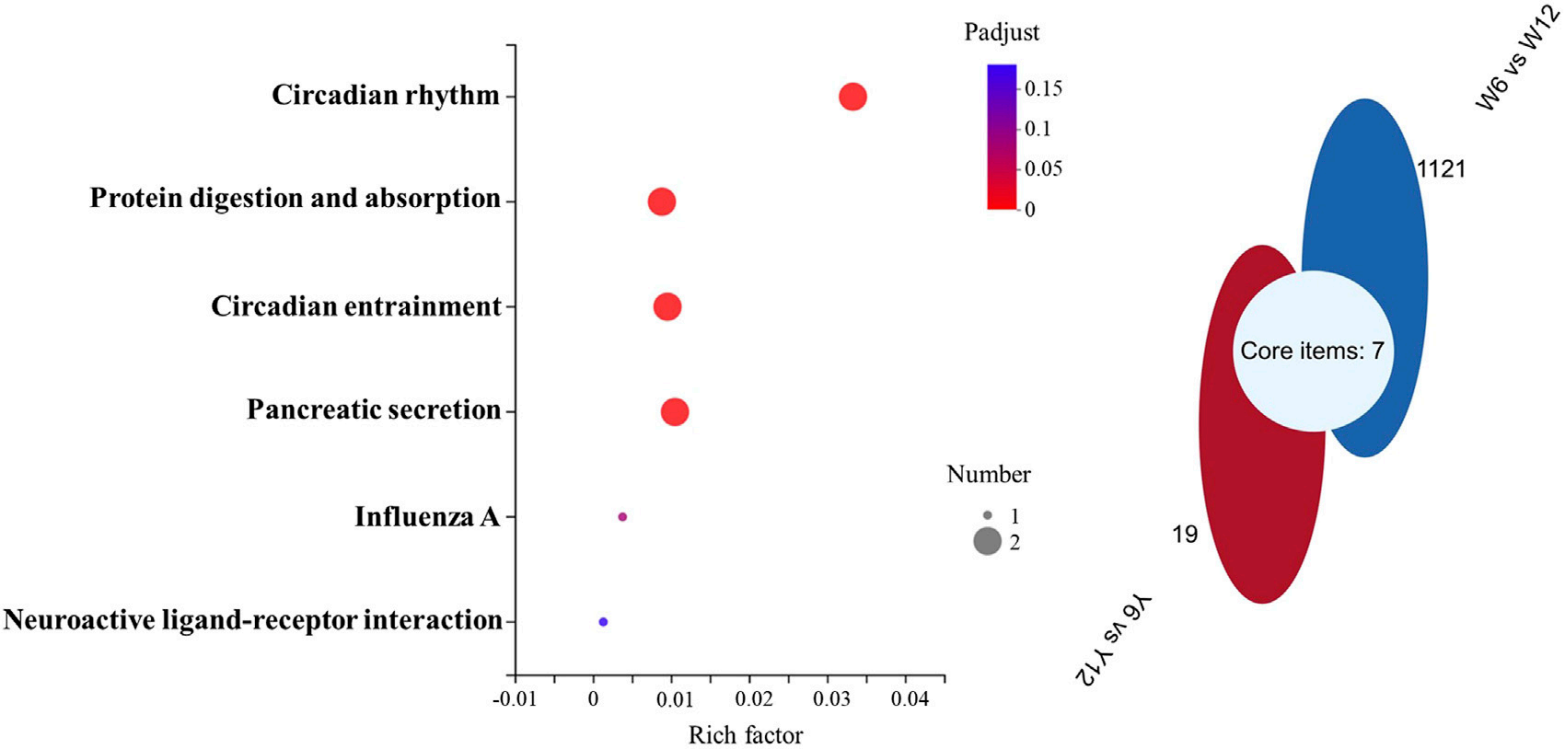
KEGG and Venn pathway analysis of the transcriptome data. Left: KEGG pathway analysis of the common genes obtained according to Venn analysis. The rich factor represents the enrichment degree, and the dot size represents the number of genes in the corresponding pathway. Right: Venn analysis of the differential genes between groups W6 and W12 (blue), and between groups Y6 and Y12 (red).

**FIGURE 6:**
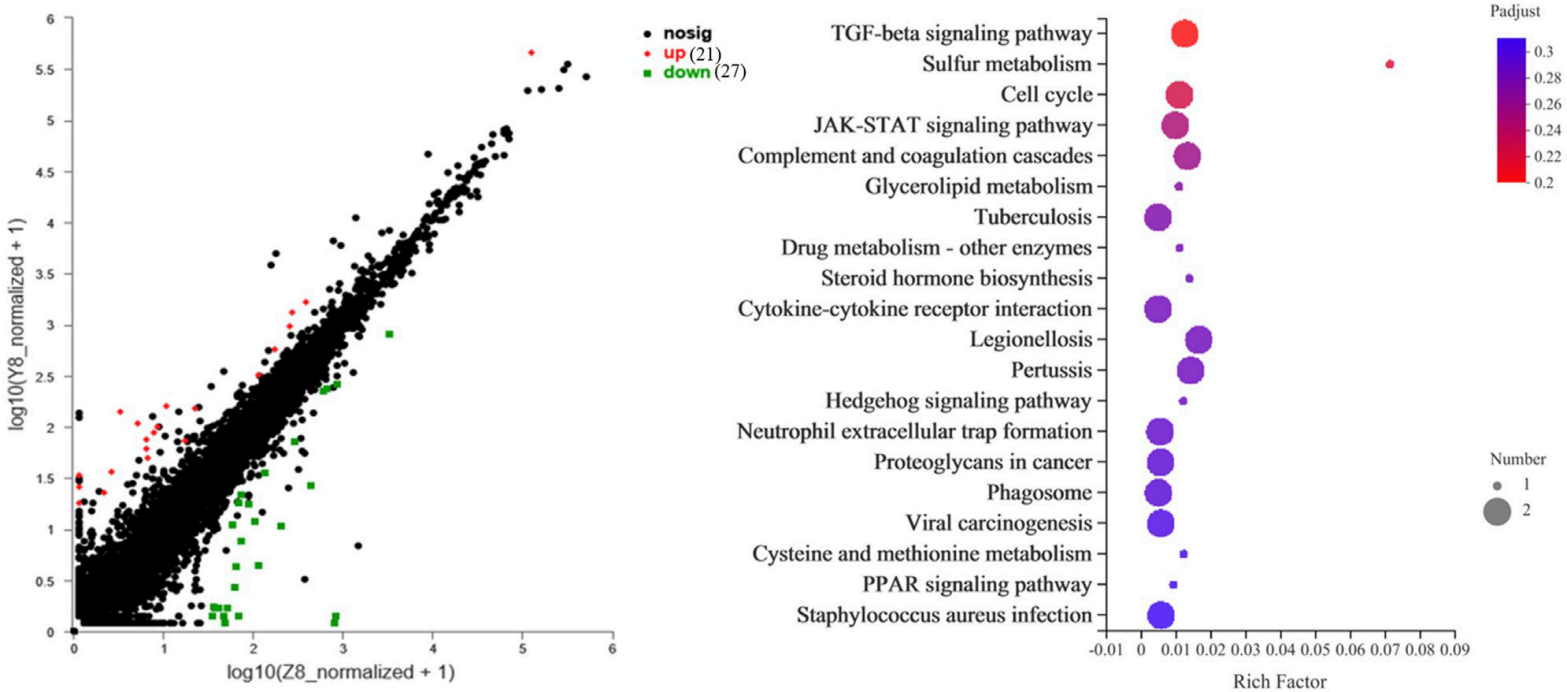
The top 20 KEGG pathway of the differential genes between W12 (control) and Y12 groups. The rich factor represents the enrichment degree, and the dot size represents the number of genes in the corresponding pathway.

Transcriptome sequencing was performed to further investigate the molecular differences in responses to glucose overload between the two strains of *M. salmoides*. First, the seven genes displayed in Supplementary Table S2 were randomly selected and measured to validate the reliability of the transcriptome. The data reveal that the expression of all genes was consistent with the results of the transcriptome analysis (Supplementary Figure S1). Furthermore, the differential genes were first screened between groups Y6 and Y12 and between groups W6 and W12. Venn analysis was performed to identify the common genes. As shown in Figure 5, seven common genes (period 1b; kallikrein-11; solute carrier family 25 member 48; period 3; f-box and leucine-rich repeat protein 3; perforin-1; carboxy-peptidas A5) were identified, and these genes were the most enriched in the “circadian rhythm” pathway. Moreover, 48 differentially expressed genes (Supplementary Table S3) between groups W12 (control) and Y12 were identified. Considering the rich factor and the number of genes enriched, the “TGF-beta signaling” and “cell cycle” pathways were the predominant signaling pathways using KEGG analysis (Figure 6). Among the differentially expressed genes, actin-associated protein, serine/threonine-protein kinase NIMI, insulin-like growth factor-binding protein 1, glycerol kinase, and dehydrogenase/reductase (SDR family) member 9 were the top five upregulated genes; while interferon-inducible GTPase 5, putative selection and upkeep of intraepithelial T-cell protein 1 homolog, coiled-coil domain-containing protein 22-like, drebrin 1, and cytochrome P450 26B1 were the top five downregulated genes (Supplementary Table S3).

## Discussion

The plasma glucose level data showed that the W strain fish required 12 h to return to baseline (basic-0 h), whereas the Y strain fish required less than 12 h. Even without statistical differences in baseline conditions (basic-0 h), the plasma glucose in strain Y was markedly lower than that in the W strain from 6 h onward, indicating that the Y strain has better glucose tolerance than the W strain. Similar abilities to metabolized glucose have also been reported in two strains of *O. mykiss* (Kamalam et all., 2012). As a hypoglycemic hormone, insulin was secreted in both strains of *M. salmoides* to resist the high glucose flux before plasma glucose could return to its baseline, as has been reported for the blunt snout bream (*Megalobrama amblycephala*) (Xu et al., 2018), *C. gibelio* (Jin et al., 2017), and *O. mykiss* (Jin J. et al., 2014). We also found that the Y strain produced more insulin than the W strain at 0 and 6 h despite both having equal basal insulin levels. This indicated that the Y strain could reduce plasma glucose more effectively through insulin.

Synthesis of TG is one of the strategies used by fish to deal with glucose load, and the incorporation of 14C-glucose in hepatic lipids increased when experimental *O. mykiss* was fed carbohydrate-rich diets (Brauge et al., 1995). Similarly, in *M. salmoides*, we found that the Y strain’s plasma and liver TG content increased and reached the maximum at 12 h, at which time the plasma glucose had returned to its baseline. Meanwhile, the expression of hepatic and muscular *fas*, *lxr-1*, and *srebp* genes that are related to TG synthesis, and the plasma content of VLDL-CH that transports excess TG were correspondingly the highest. The W strain exhibited different responses, including a stable TG content and unaffected *fas* and *lxr-1* transcription. Lower levels of VLDL-CH observed at 24 h may be due to the low TG content, although the difference was not statistically significant. These data suggest that there is a divergence in glucose overload response between the two strains, which is primarily reflected in the TG synthesis.

Glycogen is the main storage form of carbohydrates and is mainly stored in the liver and muscle (Dabrowski and Guderley, 2002). Hepatic and muscle glycogen levels were higher in fish that were fed a carbohydrate-rich diet than in fish that were fed a carbohydrate-free diet (Hemre et al., 2002; Polakof et al., 2012). In this study, we observed that hepatic glycogen and muscular glycogen increased first and then declined and peaked at 6 and 12 h in both strains, which was consistent with the change in plasma glucose levels. The hepatic and muscular *gs* genes involved in glycogen synthesis displayed the same trend, suggesting that the two strains of fish stored excess glucose as glycogen. Notably, muscular glycogen was still higher at 24 h (lower plasma glucose than the baseline). It has been reported that different to that in the liver, muscle glycogen liberation is closely related to the need for ATP rather than the synthesis of metabolic intermediates. Therefore, the muscle glycogen is barely breakdown to maintain plasma glucose without a burst of exercise (Dabrowski and Guderley, 2002).

Glycolysis is the only pathway involved in glucose breakdown in fishes (Cowey and Walton, 1989). Here, the expression of glycolytic pathway genes (*gk*, *pfk*, and *pk*) fluctuated with plasma glucose level recovery time in the Y strain’s liver and muscle, indicating that glycolysis reduced plasma glucose, which was similar to the results reported by (Enes et al., 2011). Interestingly, the W strain hepatic *gk*, *pfk,* and *pk* were stable within 12 h (the time required for plasma glucose recovery) and were suppressed in the muscle even when plasma glucose was still high, suggesting that glycolysis was not regulated by plasma glucose levels. This has also been observed in Nile tilapia (*Oreochromis niloticus*) (Boonanuntanasarn et al., 2018).

Glycolysis is followed by the Krebs cycle to fully oxidize glucose to CO_2_ and H_2_O, and to produce ATP in fish, as in mammals (Dabrowski and Guderley, 2002). In this study, hepatic *icdh-1* and *kgdc* which are involved in the Krebs cycle increased and reached a maximum at 12 h in the Y strain, which aligned with the change in glycolytic genes. However, in the muscle, the two genes were not affected, which may have been caused by the somewhat different regulatory mechanisms between liver and muscle carbohydrate metabolisms. Muscles are primarily governed by the demand for ATP during exercise (Dabrowski and Guderley, 2002). In the W strain’s liver and muscle, although the two genes were highly expressed before plasma glucose recovery (12 h) compared to after plasma glucose recovery, they (increased first after declining) were not consistent with the change in glycolysis genes (decreased gradually). There are likely other pathways that provide acetyl-CoA—the raw material for the Krebs cycle, but further research to determine this is required.

Gluconeogenesis is the process by which glucose is synthesized from non-glycosidic substrates (Enes et al., 2009). Although study has shown that the gluconeogenesis pathway is inhibited in fish that were fed a carbohydrate-rich diet (Panserat et al., 2002), the genes involved in gluconeogenesis including *pepck* and *g6p*, were promoted in the livers and muscles of both strains to face high glucose flux. This is consistent with many studies, in which the gluconeogenesis has been demonstrated to independent of plasma glucose levels (Enes et al., 2011; Boonanuntanasarn et al., 2018). The regulation of gluconeogenesis is diverse among different species and can be species-specific. Most surprisingly, the *fbp* gene decreased in the Y strain, which requires further research.

To further investigate the similarities and differences between the glucose overload response mechanism of the two strains of *M. salmoides*, liver samples from 6 to 12 h, which are the core times for plasma glucose recovery, were used for transcriptome analysis. According to the data, period 1b, kallikrein-11, solute carrier family 25 member 48, period 3, f-box and leucine-rich repeat protein 3, perforin-1, and carboxy-peptide A5 were identified as the common genes that functioned to resist hyperglycemia in the two strains. In mammals, these genes are implicated in insulin resistance, regulating plasma glucose levels, and glucose tolerance (Montanari et al., 2005; Xu et al., 2011; Yang et al., 2013). Moreover, the results showed that these common genes were mainly enriched in the circadian rhythm pathway, which is a biological timekeeping system that *via* physiology and behavior regulates the body’s material (e.g., glucose, lipids, cholesterol, and bile acids) metabolism (Ferrell and Chiang, 2015). Compared to those on mammals, such studies on fish have been limited.

For the differences underlying the glucose overload response mechanism between the two strains, only 48 differential genes were found, indicating that although the two strains of *M. salmoides* exhibited diverse responses to a high glucose load after artificial breeding, most of the similarities remained. Among the differential genes, insulin-like growth factor-binding protein and glycerol kinase were upregulated in the Y12 group. Previously, glycerol kinase was correlated with glycogen synthase gene expression in mice (*Mus musculus*) (Rahib et al., 2007), and insulin-like growth factor-binding protein had been demonstrated to reduce the hepatic glucose production rate and increase peripheral glucose uptake in human (*Homo sapiens*) (Simpson et al., 2004). Therefore, stimulation of these two genes in the Y strain would be expected. Among the top five downregulated genes, two genes (interferon-inducible GTPase 5 and putative selection and upkeep of intraepithelial T-cells protein homolog) involved in immunity were identified. In fish, excess glucose can negatively affect immunity (Vielma et al., 2003; Liu et al., 2012), which may account for the relatively high expression of immune genes in the W strain. Furthermore, genes associated with substance metabolism (dehydrogenase/reductase (SDR) family member 9, cytochrome P450 26B1), signal transduction (serin/threonine-protein kinase NIMI), cell migration and growth (actin-associated protein, drebrin 1), and protein structure (coiled-coil domain-containing protein 22-like) were also identified as candidate genes regulating glucose homeostasis between the two strains of *M. salmoides*. Although there is limited information regarding the effects of these genes on carbohydrate metabolism, it warrants further study. On the other hand, the TGF-β signaling pathway and cell cycle have been considered the main pathways controlling the differences in glucose overload response between the two strains in terms of rich factors and rich numbers, and have also been widely reported to be involved in glucose metabolism in mammals (Jin et al., 2003; Grabiec et al., 2014).

## Conclusion

**FIGURE 7:**
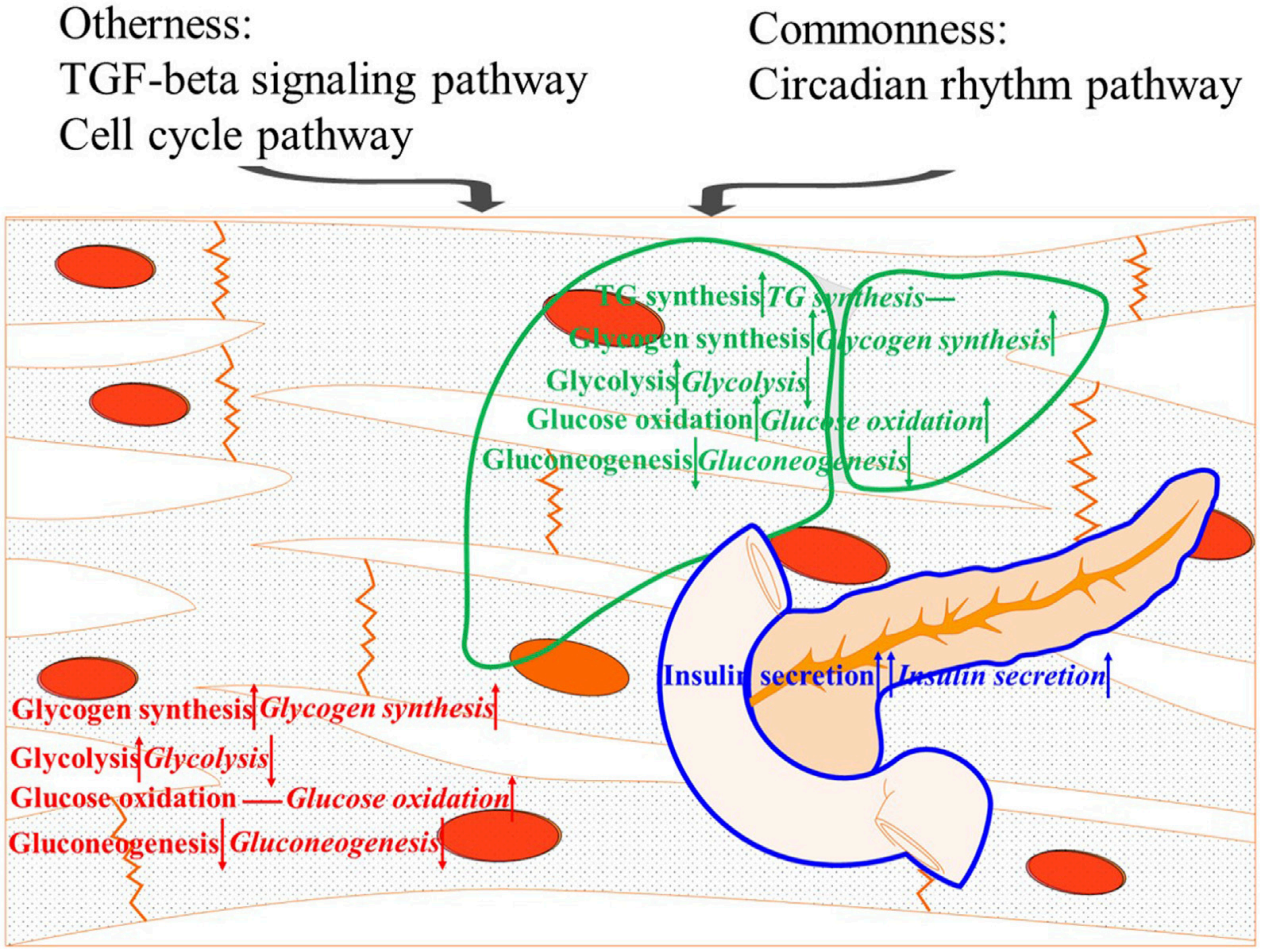
Summary diagram of the results. The upward arrow, downward arrow, and horizontal line means a positive effect, a negative effect, and no effect on resisting hyperglycemia, respectively.

This study investigated the similarities and differences in glucose overload response between two strains of *M. salmoides*. The popularly selected strain (Y strain) showed better glucose tolerance than the wild-type strain (W strain). In detail, the Y strain fish were more effective at resisting hyperglycemia by secreting insulin, synthesizing TG, and *via* glycolysis, although the oxidation of glucose in the muscle was inferior to that of the W strain fish. Differences in tissue glycogen were also found in response to glucose load that were mainly reflected in the muscle glycogen, which is more difficult to break down than liver glycogen. Gluconeogenesis was unregulated by blood glucose levels seemed to be one of the causes of intolerance to glucose in *M. salmoides*. Moreover, employing the circadian rhythm was a common strategy for controlling glucose overload in the two fish strains. However, the TGF-β and cell cycle pathways played an important role in the better glucose tolerance of the Y strain (Figure 7). These results provide new targets for further improving the poor capability of glucose regulation in *M. salmoides*.

## Data Availability

Data is contained within the article or Supplementary Materials. The transcriptome data presented in the study are deposited in the NCBI repository, and the accession number is PRJNA874753.
