# Supplementary material for: Different responses to glucose overload between two strains of largemouth bass (Micropterus salmoides)
Source: Front Physiol. 2022 Sep 28;13:1010633. doi: 10.3389/fphys.2022.1010633 (PMC9554351; doi:10.3389/fphys.2022.1010633)

**Table S1.** Six databases used for sequence annotation

| Database | Web site |
| --- | --- |
| National Center for Biotechnology Information NR database | <ftp://ftp.ncbi.nlm.nih.gov/blast/db> |
| Swiss-Prot protein database | <http://web.expasy.org/docs/swiss-prot_guideline.html> |
| Pfam database | <http://pfam.xfam.org/> |
| Clusters of Orthologous Groups (COG) of proteins database | <http://www.ncbi.nlm.nih.gov/COG/> |
| Gene Ontology (GO) database | <http://www.geneontology.org> |
| Kyoto Encyclopedia of Genes and Genomes (KEGG) database | <http://www.genome.jp/kegg/> |

**Table S2.** Primers used to validate the differential genes

| Gene | forward primers (5’-3’) | reverse primers (5’-3’) | annealing | product |
| --- | --- | --- | --- | --- |
| cytochrome P450 26B1 | AAGAGGGGCGCTTCAACTAC | GCCAGCTCAATAGCGAGGAT | 60℃ | 103 bp |
| drebrin 1 | GCGACGATGACAACGAAGTG | AGTCCTCTTTGATGGGCGTG | 60℃ | 90 bp |
| insulin-like growth factor-binding protein 1 | TGCCAAGCCCATTTCAGTCT | GCTGTGTTGTTTCGTGCTGT | 60℃ | 83 bp |
| glycerol kinase | ATGTTTGGGACGGTGGACTC | GGGAGGATTTCCATCGGGAC | 60℃ | 173 bp |
| glypican 1b | CCTCCCCTGCATCACCATTT | CTTTCCGACTCTCCCCAACC | 60℃ | 122 bp |
| cyclin-dependent kinase inhibitor 2A/B | AGTGTGTACCCTGCCTTTCG | ACCGCTTATCCTGCGTACAG | 60℃ | 79 bp |
| general transcription factor IIH subunit 5-like | TAGCACAGGAAACAACCGCA | TTTGAGCCCGGTTAGCTTGT | 60℃ | 186 bp |

**Table S3**. Differentially expressed genes between groups W12 (control) and Y12. Significant genes were selected based on p<0.05, and |log2FC|≥1. The *P* values were collected using Benjamini/Hochberg method.

| Number | Gene id | Gene description | W12(control) *vs* Y12 |
| --- | --- | --- | --- |
| 1 | gene-si_ch211-218o21.4 | actin-associated protein | up |
| 2 | gene-LOC119910744 | interferon-inducible GTPase 5 | down |
| 3 | gene-LOC119910828 | putative selection and upkeep of intraepithelial T-cells protein 1 homolog | down |
| 4 | gene-LOC119914364 | coiled-coil domain-containing protein 22-like | down |
| 5 | gene-LOC119910849 | drebrin 1 | down |
| 6 | gene-LOC119905076 | cytochrome P450 26B1 | down |
| 7 | gene-LOC119904966 | uncharacterized LOC119910828 | down |
| 8 | gene-LOC119884384 | uncharacterized LOC119884384 | down |
| 9 | gene-si_ch211-22d5.2 | serine/threonine-protein kinase NIM1 | up |
| 10 | gene-LOC119887259 | insulin-like growth factor-binding protein 1 | up |
| 11 | gene-LOC119908634 | glycerol kinase | up |
| 12 | gene-dbn1 | uncharacterized LOC119910849 | down |
| 13 | gene-LOC119913165 | uncharacterized LOC119905076 | down |
| 14 | gene-LOC119909550 | general transcription factor IIH subunit 5-like | down |
| 15 | gene-cntfr | ciliary neurotrophic factor receptor, transcript variant X3 | down |
| 16 | gene-LOC119891503 | uncharacterized LOC119891503, transcript variant X1 | down |
| 17 | gene-LOC119898167 | dehydrogenase/reductase (SDR family) member 9 | up |
| 18 | gene-selenon | selenoprotein N, transcript variant X1 | down |
| 19 | gene-LOC119899454 | complement C3-like, transcript variant X1 | down |
| 20 | gene-mb | myoglobin, transcript variant X1 | down |
| 21 | gene-LOC119903459 | uncharacterized LOC119908634 | up |
| 22 | gene-LOC119900264 | indian hedgehog B protein-like | down |
| 23 | gene-dhrs9 | uncharacterized LOC119898167 | up |
| 24 | gene-pcsk1nl | proprotein convertase subtilisin/kexin type 1 inhibitor, like | down |
| 25 | gene-ahcyl1 | adenosylhomocysteinase-like 1, transcript variant X1 | up |
| 26 | gene-arl13a | ADP-ribosylation factor-like 13A, transcript variant X1 | up |
| 27 | gene-LOC119888725 | proximal tubules-expressed gene protein-like | down |
| 28 | gene-LOC119890594 | EMILIN-1-like | up |
| 29 | gene-il23r | interleukin 23 receptor, transcript variant X1 | up |
| 30 | gene-LOC119884984 | cadherin-12-like | down |
| 31 | gene-cdkn2a_b | cyclin-dependent kinase inhibitor 2A/B (p15, inhibits CDK4) | up |
| 32 | gene-LOC119917147 | arginine vasopressin-induced protein 1-like, transcript variant X1 | up |
| 33 | gene-slc2a15a | solute carrier family 2 member 15a, transcript variant X1 | down |
| 34 | gene-prg4b | proteoglycan 4b, transcript variant X1 | down |
| 35 | gene-LOC119898429 | inositol monophosphatase 3-like | up |
| 36 | gene-LOC119912955 | type-2 ice-structuring protein-like | up |
| 37 | gene-LOC119910783 | integrin beta-2-like | down |
| 38 | gene-LOC119907471 | Ig kappa chain V-III region MOPC 63-like | up |
| 39 | gene-LOC119908630 | exostosin-1-like, transcript variant X1 | up |
| 40 | gene-LOC119911593 | uncharacterized LOC119911593 | down |
| 41 | gene-tnnc1b | troponin C type 1b (slow), transcript variant X6 | down |
| 42 | gene-gpc1b | glypican 1b | down |
| 43 | gene-tsnaxip1 | translin-associated factor X interacting protein 1, transcript variant X1 | up |
| 44 | gene-LOC119912963 | uncharacterized LOC119912963 | up |
| 45 | gene-haus2 | HAUS augmin like complex subunit 2 | up |
| 46 | gene-LOC119886785 | dynein heavy chain 12, axonemal-like | down |
| 47 | gene-LOC119882035 | transcription factor Dp-1-like | up |
| 48 | gene-ugt5d1 | UDP glucuronosyltransferase 5 family, polypeptide D1 | down |

**Figure S1**. PCR validation of differential genes (n=12). 1-7: cytochrome P450 26B1, drebrin 1, insulin-like growth factor-binding protein 1,

glycerol kinase, glypican 1b, cyclin-dependent kinase inhibitor 2A/B, general transcription factor IIH subunit 5-like.


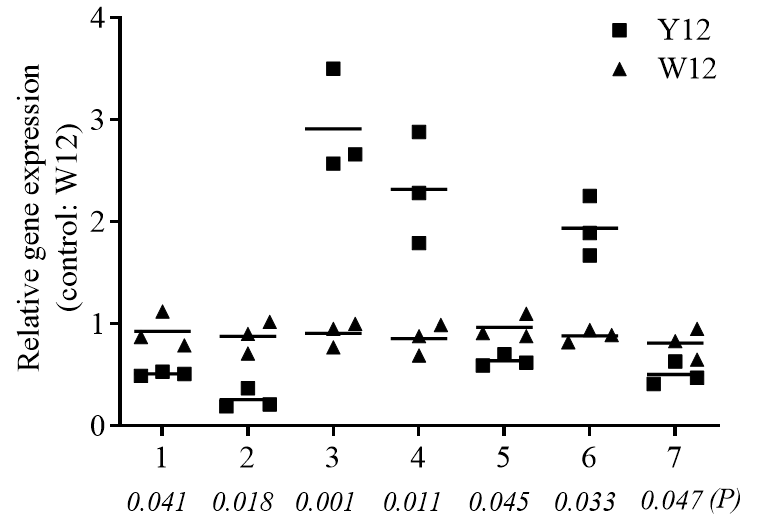

Supplement: Supplementary file 1 [file DataSheet1.docx]
